# Assessing the generalisability of a multicentre qualitative dementia research: the experience and challenges faced by the MinD project in Europe

**DOI:** 10.12688/openreseurope.13700.2

**Published:** 2021-09-09

**Authors:** Jennifer N.W. Lim, Kristina Niedderer, Isabelle Tournier, Rosa Almeida, Dew Harrison, Vjera Holthoff-Detto, Geke Ludden, Thomas van Rompay, Mascha van der Voort, Aleksandra Galansinska, Tina Smith, Raquel L. Lasada, Yolanda A. Bueno, Diana Druschke, Berit Ziebuhr, Michele Zanasi

**Affiliations:** 1 Faculty of Education, Health and Wellbeing, University of Wolverhampton, Wolverhampton, WV1 1LY, UK; 2 Manchester School of Art, Manchester Metropolitan University, Manchester, UK; 3 INTRAS, Valladolid, Spain; 4 Faculty of Arts, Business and Social Sciences, University of Wolverhampton, Wolverhampton, UK; 5 Alexianer Krankenhaus Hedwigshoehe, St. Hedwig Kliniken, Berlin, Germany; 6 University Hospital Carl Gustav Carus and Carl Gustav Carus Faculty of Medicine, Technical University of Dresden, Dresden, Germany; 7 Department of Design, Production and Management & Department of Communication Science, University of Twente, Enschede, The Netherlands

**Keywords:** People living with dementia, generalisation, multicentre design, interdisciplinary, qualitative study, cross-cultural, Europe

## Abstract

**Background**: Generalisation of findings is an important aspect of research and essential for evidence-based practice. While generalisation is common in quantitative research, there is a lack of generalisability in qualitative research. This paper presents the experience and challenges faced by the Designing for People with Dementia (MinD) project in meeting the requirements to strengthen the generalisation of findings on the lived experience of people living with dementia and their engagement to co-create designs to empower their everyday living.

**Methods**: Polit and Beck (2010)’s strategies to generalise qualitative findings were applied: (1) replication in sampling; (2) replication of studies; (3) meta-synthesis of findings; (4) reflexivity and conceptualization; (5) immersion with the data; and (6) thick description.

**Results**: While it is possible to increase the generabilisabilty of qualitative evidence through the replication of the sampling to attain a large, heterogeneous sample in different and multiple contexts and environments; implementation of sound and robust research; conducting in-depth analysis and interpretation collaboratively for emergent themes; and meeting the thick description requirement, there are challenges that the project team faced in implementing some of the Polit and Beck’s strategies because of the condition, namely dementia, that our participants are having. Other challenges faced were: the language and cultural diversity in the team; diverse  work and organisational procedures; and the inter-disciplinary differences relating to the methods of enquiry, approaches and techniques to conduct research. These challenges will need to be identified and addressed at the start of the project with a strong leadership to ensure a seamless journey to complete the project successfully. Trust between the researchers and participants, and time to build this trust are critical to recruitment and participation in the study; these factors are of utmost important in research involving participants with condition such as dementia.

## Plain language summary

This paper describes the use of the multi-centre design by a group of researchers - with different expertise from different discipline of study - to apply the six strategies proposed by Polit and Beck to strengthen the generalisability of their research findings in the population living with mild to moderate dementia in Europe. The authors also presented the challenges they faced in their attempts to generalise their findings. They concluded that it is possible to make claim for generalisation of findings in qualitative research which involved a unique population group namely people living with dementia.

## Introduction

Generalisation of findings is an important aspect of research and essential for evidence-based practice. To be useful, research findings have to have some relevance for other settings and people outside of the context studied *.* Generalisable results are used to inform and shape policy making, and the design and development of interventions to enable wider application, e.g. in other settings or population groups. The ability to generalise is a key criterion for quantitative research, and it is also important for qualitative research ^1– 3^. 

Transferability of findings across settings is common in qualitative research but generalisation remains an issue that has been widely discussed. However, the general consensus among researchers is that generalisation should be a legitimate concern for qualitative researchers and that they should engage with the generalisability of their results ^4– 10^. Ignoring generalisation in qualitative research can prevail the assumption that this method is limited by the lack of its ability to generalise; leading to it not being taken seriously in decision-making ^11^.

Firestone (1993) described three models that are used to make claims for the generalisation of research findings: (1) extrapolation from sample to population; (2) extrapolation using a theory; and (3) case-to-case translation. Respectively, these models are better known as: (1) statistical, also known as naturalistic and representational generalisation; (2) theoretical or analytic generalisation; and (3) inferential generalisation ^12^. The statistical generalisation model is applied in quantitative research while naturalistic and representational generalisation are amenable for qualitative research. Naturalistic and representational generalisation can be reached on the basis of recognition of similarities and differences between two groups or cases. Inferential generalisation, also referred to as *transferability* ^13^ or *case to case* generalisation ^14^, is about the extent to which the results are transferable to other settings. To judge the generalisability of research findings, researchers have to provide the evidence for their claims that readers need to critically assess for quality and usability of the research, particularly on the validity and reliability of the research, and in qualitative research, this is called thick description of study or rich data ^15^. This evidence includes the methodological and scientific rigour of the research as well as the research context and process.

Apart from the requirement of rich data, generalisability of research is also dependent on whether the research was conducted in a sound and robust manner, i.e. meeting two standards - validity and reliability. In general, validity is an indication of the soundness of a research study and is determined by the design and the methods employed to conduct the research; whereas reliability is about the consistency of the methods used, and whether the study is replicable to produce the same results. In qualitative research, other terms are used interchangeably to describe validity and reliability. For validity, researchers have used ’quality’,’ rigour’, ‘well-grounded’ and ‘trustworthiness’; and for reliability, ‘credibility’, ‘neutrality’, ‘confirmability’, ‘consistency’ ‘dependability’, ‘sustainable’, ‘applicability ‘ and ‘transferability’ ^15– 18^. The discussion of the different terms and the rigour in which a qualitative study is conducted is essential to ensure the credibility of findings and strategies to reach it are matter of ongoing discussion ^19^. A sound and robust study will strengthen the claims for generalisation.

Numerous strategies have been recommended to strengthen the generalisability of qualitative research findings. Polit and Beck ^20^ suggested six steps to meet both the validity and reliability standards of research: (1) replication in sampling, using techniques such as purposive sampling and maximum variation sampling to increase heterogeneity in the samples and achieve data saturation in a large sample; (2) replication of studies to confirm the findings (concepts, relationships, patterns and successful interventions) in multiple contexts and times, and with different types of people, as this will strengthened the confidence in the validity and application of the evidence; (3) meta-synthesis of findings about a phenomenon from multiple qualitative studies and techniques; (4) reflexivity and conceptualisation by the researcher about the consequences of applying the findings to the new context; (5) immersion with the data; and (6) thick description. Lewis and Ritchie ^15^ also focused on meeting the research standards; they provided a list of questions about the study design, methods, and the congruence between the sending and receiving contexts involved in the generalisation, and emphasised the need for thick description of the study. Gheondea-Eladi ^21^ showed that qualitative research is generalisable when the appropriate sampling, coding and data analysis methods are employed.

The strategies proposed by Polit and Beck ^20^ are theoretically valid and should guide every research, however, in practice these steps may be more complex. This paper reviews the experience and challenges faced by the MinD project ^22^ in meeting Polit and Beck’s requirements to deliver the generalisation of its findings, and to reflect on the application of Polit and Beck’s six steps in practice.

## Methods

### Ethical approvals

Ethics approval for the study was obtained by each of the partners in line with national and European regulations and requirements: UK: University of Wolverhampton and Manchester Metropolitan University ethics boards, ethics reference no. 2018/19:18 (UW) and Ethos 5521 (MMU); Germany: Krankenshaus Hedwigshöhe ethics reference no. Eth-30/16; The Netherlands: University of Twente, ethics reference no. BFD-BMS/2016-JR; Spain: INTRAS: Reference letter 26/01/2016.

### Qualitative dementia research: Designing for People with Dementia project (MinD)

Qualitative studies provide an insight into the lived experience of people with dementia (PwD) as well as demonstrating that PwD can participate in research ^23– 28^. These studies tend to use small sample sizes and often lack the rich details for readers to judge the quality of research and the extent to which their findings are generalisable. A number of meta-synthesis reporting the lived experience of people with dementia are available, but these reviews did not provide sufficient contextual information required by readers to critically assess and decide on their application and generalisation ^26, 29– 31^. A preliminary search revealed that there is not yet a multicentre qualitative study exploring the lived experience and needs of PwD. The project *Designing for People with Dementia: Designing for mindful self-empowerment and social engagement* - abbreviated as MinD - fulfilled this gap in research by implementing a multi-national and interdisciplinary qualitative study engaging people with mild to moderate dementia (PwD) through a co-creation approach in developing designs appropriate to their needs, wants and preferences. 

Funded by the European Union’s Horizon 2020 MSCA RISE programme, the MinD project, a 4-year project (March 2016 – February 2020), aimed to help PwD engage in social contexts to improve and maintain their psychosocial wellbeing through design and mindfulness ^32^. It was based on the observation that social engagement is rarely addressed by psychosocial and design interventions, whereas social contact and enjoyment of activities appear as key aspects of the unmet needs for people living with dementia ^33, 34^. The choice of a qualitative approach for the MinD project was justified by two mains aspects: a) the need for rich and contextualised data regarding social participation needs and issues encountered by PwD, and b) data collection format adapted to the co-design and co-production approach deployed throughout the project to identify needs and design solutions to better manage dementia in everyday living. The COREQ guideline was applied to deliver a rigorous study throughout the project ^35– 37^.

### A multicentre design and interdisciplinary approach

Multicentre design is a practical approach to accumulate sufficient numbers of diverse participants in a substantially shorter period of time than could be effected by a single centre or study site ^22, 38– 40^. This means a greater number of environments and contexts from which participants are recruited, offering a more representative sample of the target population of the study and allowing for replication of sampling to achieve heterogeneity and maximum variation; thus increasing the strength of generalisation.

An interdisciplinary approach benefits research in many ways. Team members from different disciplines can share skills, expertise, knowledge and experience throughout a project. Lee and colleagues ^41^ contend that an interdisciplinary team enables developing a richer and more complex understanding. Furthermore, collaborative working can improve the quality of the research and its rigor, through enhanced group reflexivity and triangulation of results by researchers with diverse backgrounds.

The multicentre MinD project applied an interdisciplinary approach; it’s team was comprised of researchers and practitioners from three major disciplines, namely design, health, and computing, including designers, architects, programmers, psychologists, gerontologists, healthcare practitioners and public health researchers – working in 18 institutions in 8 countries (the UK, Germany, Luxembourg, the Netherlands, Spain, Italy, Russia and Australia) as well as partner groups of people with lived experience of dementia such as the Groups of Experts by Experience (GEEs) and Patient and Public Involvement group (PPI) ^32^. Regular two-week exchanges were implemented in each of the partner countries to facilitate the interdisciplinary and iterative research process of the project, and there were a total of 49 exchange visits involving 75 researchers over the 4-year period.

## Results

The MinD project conducted the research in three phases: Phase 1 - needs assessment with PwD and caregivers; Phase 2 - development of mindful design interventions (designs) through co-design workshops with PwD, caregivers, service users, GEEs and PPI, using the data collected in the first phase. Three prototypes (“Good Life Kit”, “This is Me” and “Let’s me up”) were developed and further information of these can be found on the MinD website ^32^. The final Phase 3 was the evaluation of the design prototypes with PwD, caregivers and service users. Qualitative data were collected at two points in this project, i.e in Phases 1 and 3, in four countries (Germany, Spain, the Netherlands and the UK), and the findings are reported elsewhere ^36, 37^.

Following the ethical approval in each country, recruitment of participants took place with the support of the healthcare professionals and care workers whom the PwD were familiar with, and during data collection, the capacity to participate was continuously monitored ^42^. The experience of the MinD project in applying Polit and Beck’s generalisation strategies are described below.

### Replication in sampling

Various purposive sampling strategies that involve deliberate replication to promote both analytic generalisation and transferability can be used to replicate sampling, namely, maximum variation sampling, critical case sampling and deviant case sampling ^20^. For the MinD project, we applied the maximum variation sampling strategy to recruit people living with mild to mid-stage dementia including people with mild cognitive impairment (MCI) who had the capacity to consent to participation.

A total of 57 people (41 PwD and 16 caregivers) participated in Phase 1 (the needs assessment) ( Table 1), while 65 (51 PwD and 14 caregivers) people participated in Phase 3 (evaluation of design prototypes) ( Table 2). The PwD and caregivers who participated in the study came from a mixed socio-economic and cultural background in all the study sites, spoke diversed languages, and were aged between 50 and 80 years old. They were from Caucasian backgrounds as there were few PwD from minority ethnic backgrounds attending the memory clinics, support groups in the study sites.

**Table 1.** Phase 1 - Needs assessment (characteristics by study site).

DETAILS	GERMANY	SPAIN	NETHERLANDS	UK
**Date of** **the needs** **assessment**	September – November 2016	March 2017	June 2017	No data collection at this phase in the UK. This UK site was used for design and development of the protoypes with the PPI group in Nottingham
**Number of** **participants who** **completed the** **study**	24 participants (6 PwD and 18 caregivers)	15 participants (9 PwD and 6 caregivers)	18 participants (14 PwD and 4 caregivers).	
**Methods of data** **collection**	Individual interviews (6 PwD and 10 caregivers ; 1 focus group (8 caregivers), Design probes in 4 groups of 3 PwD each (12 PwD)	Individual interviews (6 PwD), Focus group (6 caregivers); Design probes (3 Pwd)	Interviews (9 pwd and 4 caregivers) Design probes (5 PwD)	
**Settings**	Day memory clinic, Department of Psychiatry, Psychotherapy and Psychosomatics or at their homes in Berlin (individual interviews) Focus groups with PwD and caregivers in Alzeimer Association groups Dresden	Specialised Memory clinic for PwD, Volladolid, Northern Spain	Homevisits	

**Table 2.** Phase 3 - Evaluation (characteristics by study site).

DETAILS	SPAIN	NETHERLANDS	UK	GERMANY
**Date of the** **evaluation**	Feb – March 2019	April 2019	June 2019	July 2019
**Number of** **participants who** **completed the** **evaluation**	42 participants (14 pwd, 14 MCI, 14 caregivers)	4 PwD	7 PwD	12 PwD
**Method of data** **collection**	4 focus groups (total participants = 42)	Individual interviews	Focus groups	Individual interviews
**Setting**	Memory clinic in Volladolid, Northern Spain	Participant’s home (3 participants; Community care home (1 participant), Enschede	Day care centre where people with dementia meet weekly for social activities, Solihull, England	Day memory clinic, Department of Psychiatry, Psychotherapy and Psychosomatics, Berlin

### Replication of studies

According to Shadish and colleagues ^43^, validity and applicability of concepts, relationships, patterns and successful interventions will be strengthened if these can be confirmed in multiple contexts, varied times and with different types of people. Deliberate replication of studies can be used as a means to confirm findings and increase generalisation ^20^.

For Phase 1 of the MinD project, data were collected to assess PwD’s needs, wants, preferences, lived experience, subjective wellbeing, self-empowerment and social engagement activities. In Phase 3, data were collected to evaluate the usability and fitness of two prototypes (the “Good Life Kit” and “This is Me”). At both phases, data collection happened consecutively across the study sites ( Table 1 and Table 2) - this sequential approach allowed not only for replication of the study, but importantly, lessons learned at previous site were useful for improving the process at subsequent study sites. In addition, sites offered multiple settings and contexts, namely, a neuro-psychological rehabilitation center and memory clinic in Spain; a memory disorder clinic and an old age day clinic at the Department of Psychiatry, Psychotherapy and Psychosomatics in Berlin, Alzeimer Association groups in Dresden, a community day care group held at a residential care home in the UK ; and in private homes of PwD in the Netherlands.

Apart from replication of studies in multiple contexts, varied time and a different group of participants, we also used a variety of data collection techniques to meet the needs of PwD. For example, visual cards of daily activities were developed - covering three areas of activities and daily life in line with the mindfulness framework of the project - to obtain a holistic understanding of the activities of daily living, social and leisure activities, involvement in decision-making, well-being linked to activity participation, supporting objects or devices, needs, wants and preferences of the participants. Participants took part in individual interviews and/or focus group discussions and in the Netherlands, diaries were also used to collect information of daily activities (see Table 1 for details). These approaches were applied across all the study sites and further details are discussed elsewhere ^32, 36, 37^.

Interview guides were developed for data collection; these were designed and tailored to the needs of PwD for each phase of data collection. These materials were reviewed by the project’s interdisciplinary team, and by the European Working Group of People with Dementia representative, and PPI in the UK, for accuracy, readibility and legibility. Specific guidance or protocol was also developed to ensure standardisation of data collection across the sites and training was provided to all the researchers involved in this process. The guides and protocols were translated into the appropriate language for the study site. Individual interviews were conducted iteratively to reach data saturation. Data collection typically lasted between 60 to 90 minutes for individual interviews and focus group discussions ^36, 37^.

### Immersion in the data and reflexibility

Immersion and reflexivity on the concepts and data throughout the project are pertinent to maintain high quality work; “ *The emergent efforts to ask questions of the right people (or to observe the right behaviours or events) force ongoing decisions that are, in theory at least, driven by conceptual demands of the study, and it is these efforts that contribute to analytic generalisation*” ^20^. Immersion and reflexivity of data and concepts in the project occurred at the data analysis and interpretation phase.

For the MinD project, the thematic analysis approach ^44^ was used to collect data and conduct rigorous analysis. Applying qualitative data analysis techniques, which allow for an association between deductive and inductive creation of categories ^45, 46^, the data were firstly analysed within each country. The inductive-deductive approach allowed *a priori* knowledge or categories from the literature to be explored in the interviews or focus groups, and for the generation of new categories/themes from the data until data saturation was met ^47^. The first step of this process involved carefully reading and rereading each transcript ^48^. It is an active reading, with the intention of appraising, familiarising, identifying, extracting, recording, organising, comparing, relating, mapping, stimulating and verifying. In other words, it is reading with “ *the intention of collating a synthesisable set of accounts*” ^48^. The second step was coding: at least two researchers in each site coded the data, performing a line-by-line coding. In the third step, similar to the translation work performed by Noblit and Hare ^49^, a second coding system to categorise participants’ answers was developed. It involved comparing and contrasting themes by cross-matching them between transcripts to leave a comprehensive set of key themes that fully represented the data from each study site. The fourth step involved presenting the set of key themes at the interdisciplinary project workshops to discuss, reflect and agree on a list of emerging themes. In the fifth and final step, the list of emerging themes was presented to the PwDs in several GEE and PPI groups for triangulation and confirmation. The finalised coding framework was systematically applied to all transcripts using quali.xls. The transcripts were translated from Dutch, German and Spanish language into English by a native speaker for the purpose of re-analysis and team consensus. MAXQDA 2018 was used to manage the data analysis.

### Integration of evidence

Polit and Beckargued that integration of evidence, which relies on replication of studies, is the most important development for enhancing generalisation in health care research ^20^. However they cautioned that integration of evidence through meta-synthesis of research studies as a methodology could lead to the loss of information about study contexts that limits assessment of proximal similarity and transferability of evidence. In the case of the MinD project, the interdisciplinary team and the PwD, GEE and PPI groups provided the avenue to triangulate the findings from different perspectives, thus preserving the richness as well as the contextual information of the study environments.

### Thick description or rich data of the research

Thick description of the research is required for readers to judge the generalisability of the findings. Throughout the MinD project, details were kept on all steps as a requirement of the funding scheme, as well as to demonstrate the study’s rigour and trustworthiness. Reports for each two-week secondment with relevant notes on workshops, and other relevant meetings notes, documents, reflective journals were kept on data collection, design and evaluation – these are located on the project intranet ^32^. In total, the MinD project convened 49 exchange visits and in each visit, the team discussed the research in each study site, reflective notes were then written up and uploaded onto the project intranet to share with partners.

## Discussion

A multicentre study design and interdisciplinary approach undertaken in the MinD project have made it possible to apply the Polit and Beck’s strategies to claim and strengthen the generalisability of our findings. However, the MinD team have faced a number of challenges during the journey to make the generalisation claim. For example, replication of the research in multiple sites is not a simple process because of the unique population and the different context and environment at each study site. These methodological challenges are explained in more details below.

### From unique population to repeat sampling

Many PwD can function independently for most activities, such as social events or using transport, while experiencing some changes and difficulties such as trouble in planning and organisation, remembering new information, locating objects, and some with finding less frequent words or proper nouns in conversations, noticeable to friends and relatives ^50^. Working with PwD therefore required specific adaptations to be met to fit their needs. Careful and detailed planning, together with PwD, was made from the start of the project to engage PwD in the MinD project ^51^. All these efforts and activities required time and care to build the trust with PwD in all the study sites for successful recruitment and engagement.

Unlike multicentre quantitative research which requires a controlled environment with participants sharing homogenous characteristics in multiple settings, exact replication of the qualitative research is not necessary. For qualitative research, the aim is to recruit a representative sample with maximum variation. The primary recruitment criteria for the MinD project was individuals living with mild to moderate dementia. This population is not only vulnerable but also considered as hard-to-reach due to their condition and the safeguarding policies put in place by relevant organisations. Collaborating with healthcare providers and gaining ethical approval from respective organisations, namely care homes and organisations overseeing support groups are the initial steps to researching the lived experience of people living with dementia, but these do not guarantee successful recruitment and data collection ^42^. We learnt through our journey that involving the people whom PwD trust, to recruit and collect data was a critical step to gain confidence and trust of PwD in our study. A lot of attention was given to secure involvement of health care professional, care and support staff whom PwD were familiar with to help researchers recruit and then obtained informed consent. The presence of the care workers and support staff during the study provided a safe environment for PwD to confidently participate in the research. We were not only able to successfully recruit PwD with the help from these staff, but maintained full participation from the PwD except for one participant who dropped out of the study ^42^.

Based on this experience in the first phase of the project (needs assessment), we then developed a dementia friendly protocol and participant information sheet for Phase 3 (evaluation of prototypes) to support the data collection by our team. This protocol emphasised the importance of gaining and understanding of the study with involvement from the care workers and support staff. Engaging PwD in research is therefore a process that required time and care in planning that involved the staff whom they are familiar with; a process that we have learnt to improve over time in our research journey and one that cannot be rushed.

### Replicating study conditions in multiple sites

To uphold the quality and rigour of our research, researchers received training and the data collection tool was standardised across the sites. However, we were not able to repeat the condition and data collection technique across the four sites with this population. To meet the needs of our participants, we had to implement different qualitative research techniques at different study sites. Individual interviews were conducted with participants in their home in the Netherlands and in memory clinics in Germany, while focus group discussion technique was used in Spain and the UK. We used various dementia-friendly research materials to meet the needs of PwD. A lot of time was spent working with PwD representatives in the GEE and PPI groups to develop the research materials to address memory, language and attentional difficulties. 

Allowing extra time at each study site is pertinent to allow for genuine engagement with PwD and their carers. It is important to go along at people’s individual pace and honour their preferred and applying diverse methods of engagement in conducting the research. This means navigating work through a compass that is process centred rather than solely output-led. We have to be mindful and constantly reflecting on our practice in order to create safe and compassionate spaces with all the PwD.

### Problems faced in interdisciplinary team

Interdisciplinary working in the MinD project has benefited the quality of the work through shared knowledge, experiences and expertise from different disciplines. In total, 75 researchers from 8 partner countries participated in 49 exchange visits over 4 years. However, interdisciplinary working has challenges in that it requires intense negotiation, coordination and management. Design, computing, and health also have distinctive methods of enquiry; approaches and techniques to conduct research. Designers and computing/technologies apply an abductive approach to produce a design (Tavory and Timmermans,2014) while a deductive or inductive approach is commonly employed by healthcare practitioners. These differences have implications on the focus and value framework of the MinD project and can lead to communication issue between members. As a design-led study, the MinD project naturally sought to make design interventions from a productive worldview ^52^. To enable a seamless study, agreements were reached through negotiating differences from the start of the project and throughout as necessary, emphasising the main aim and objectives of the project, managing expectations of all parties involved, and importantly, building understanding of the strengths of productive and inductive approaches and the need to produce sound and robust data. From the team perspective, diversity in background, culture and discipline means a longer time for the members to develop relationships and trust to avoid communication issues. Over time, the researchers reported that they better understood, and adapted to, cross-disciplinary, cultural and language barriers in the interdisciplinary exchange visits and that they felt they benefited and enjoyed the networking and learning about the different disciplines and their approaches.

### Logistic issues in a multicentre design

Achieving logistical parity between sites and personel in different countries was a challenge due to the particular funding scheme and the delivery through secondments or research exchanges but also due to the developmental nature of design projects. While a generic framework existed for coordinating the study in the four countries, adjustments in allocation of staffing was required with regard to available human resources of who is doing what, for what purpose, how, and where, during the project. This caused at times slight delays with data collection, and demanded good management and coordination regarding staffing. Other logistic and management tasks included the allocation of space for meetings and workshops; the development and agreement of standard protocols, procedures and guidelines for recruitment, data collection and analysis; as well as identifying technological capacity (experts and IT equipments) as needed.

During the MinD project, we have applied sound and robust methodological frameworks to conduct our study; we have developed standardised dementia friendly data collection guides and protocols for use across the four sites; we have used a variety of tools, methods, techniques, and settings to meet the needs of our participants in recruitment, data collection and evaluation; and we have applied thematic analysis to conduct a rigorous and trustworthy study, adopting an approach similar to that applied by Nowell and colleague ^53^. By following rigorous protocols to conduct the qualitative research, we also met the guidelines to plan and conduct qualitative research as stated in the Consolidated criteria for reporting qualitative research (COREQ) reporting guidelines ^35^.

The MinD approach was characterised by being international, cross-cultural, interdisciplinary, inter-sectoral, and iterative in its nature. Apart from the benefits gained through shared knowledge, ideas, and experiences from different disciplines, sectors/settings, and cultures, these differences allowed for triangulation to improve the validity of our project. Thus, applying a multicentre and interdisciplinary approach has enabled the MinD project to increase the generalisability of their findings over a single centre study and aiding their generalisability across disciplines, sectors and cultures. Facilitated through the complex pattern of 49 official exchange visits involving 75 researchers, this multicentre, interdisciplinary approach also created a community of practice for the researchers who have developed a broader and richer knowledge base, sharing their knowhow and experience with other researchers, groups with special interests and the general public through the MinD website and other channels ^32^.

To our knowledge, by applying an interdisciplinary approach in a multi-centre study, the MinD project is the first qualitative study to attempt to generalise its findings. Other strengths of our study are the involvement of PwD, through a co-creation approach throughout the project, at their pace; applying a mindfulness approach to engaging PwD and multiple data collection techniques fitting their needs; and the involvement of the healthcare professional, care worker and support staff whom the PwD were familiar with in the research which successfully retained PwD’s participation. Although we aimed for a heterogenuos sample, we only recruited Caucasian PwD. We did not manage to recruit PwD from the minority ethnic groups since they have low attendance in our study sites; our findings and prototypes therefore might have limited generalisability in this population, and further research in the minority ethnic population is needed.

## Conclusion

While it is possible to increase the generalisability of qualitative evidence through a multi-centre and interdisciplinary team approach and to use existing tools such as the COREQ, research knowledge and skills, experience of working with the target population to conduct a rigorous study, there are challenges that we faced because of the condition, namely dementia, that our participants are having, the diversity in terms of language, work culture and organisational procedures, and the inter-disciplinary differences relating to the methods of enquiry; approaches and techniques to conduct research. These challenges will need to be identified and addressed at the start of the project with a strong leadership to ensure a seamless journey to complete the project successfully. Trust between the researchers and participants, and time to build this trust are critical to recruitment and participation in the study; these factors are of utmost important in research involving participants with condition such as dementia.

## Data availability

No data are associated with this article.
