# Supplementary material for: Assessing the generalisability of a multicentre qualitative dementia research: the experience and challenges faced by the MinD project in Europe
Source: Open Res Eur. 2021 Nov 10;1:64. Originally published 2021 Jun 10. [Version 3] doi: 10.12688/openreseurope.13700.3 (PMC10446055; doi:10.12688/openreseurope.13700.3)
Supplement: Peer review history

# Peer review: Reviewer response for version 2

**DOI:** 10.21956/openreseurope.15224.r27982

**Published:** 2021-11-08

**Authors:** Jihui Lv

**Affiliations:** 1 Beijing Geriatric Hospital, Beijing, China

In the current study, the authors described the use of the multi-centre design by a group of researchers - with different expertise from different discipline of study - to apply the six strategies proposed by Polit and Beck to strengthen the generalisability of their research findings in the population living with mild to moderate dementia in Europe. The authors also presented the challenges they faced in their attempts to generalise their findings. The results are reasonable and interesting.

 I have two minor concerns.

1. In page 3, “……as this will strengthened”, it should be “will strengthen”.
2. The introduction part can be more concise and comprehensive, for example, the third paragraph and fourth paragraph unnecessarily mentioned validity and rich data repeatedly.

Is the rationale for developing the new method (or application) clearly explained?

Yes

Is the description of the method technically sound?

Yes

Are the conclusions about the method and its performance adequately supported by the findings presented in the article?

Yes

If any results are presented, are all the source data underlying the results available to ensure full reproducibility?

Yes

Are sufficient details provided to allow replication of the method development and its use by others?

Yes

Reviewer Expertise:

A specialist working at congnitive impairments.

I confirm that I have read this submission and believe that I have an appropriate level of expertise to confirm that it is of an acceptable scientific standard.

# Peer review: Reviewer response for version 2

**DOI:** 10.21956/openreseurope.15224.r27575

**Published:** 2021-09-10

**Authors:** Maznah Dahlui

**Affiliations:** 1 Centre of Population Health, Department of Social and Preventive Medicine, Faculty of Medicine, University of Malaya, Kuala Lumpur, Malaysia

I am OK with this new version. I suggest the article is accepted for indexing.

Is the rationale for developing the new method (or application) clearly explained?

Yes

Is the description of the method technically sound?

Yes

Are the conclusions about the method and its performance adequately supported by the findings presented in the article?

Yes

If any results are presented, are all the source data underlying the results available to ensure full reproducibility?

Yes

Are sufficient details provided to allow replication of the method development and its use by others?

Yes

Reviewer Expertise:

NA

I confirm that I have read this submission and believe that I have an appropriate level of expertise to confirm that it is of an acceptable scientific standard.

# Peer review: Reviewer response for version 1

**DOI:** 10.21956/openreseurope.14774.r27444

**Published:** 2021-09-06

**Authors:** Maznah Dahlui

**Affiliations:** 1 Centre of Population Health, Department of Social and Preventive Medicine, Faculty of Medicine, University of Malaya, Kuala Lumpur, Malaysia

This manuscript describes a study that had applied a standardised study protocol, of which the framework and guides for the qualitative data collection were developed via a multidisciplinary approach for a multicentre study. The study aims to explain that a study conducted as such would allow its findings to be generalised for Dementia. Polit and Beck’s criteria had been used to assess the generalisability of these qualitative research findings.  In addition, issues and challenges faced when implementing and evaluating the multicentre studies had been described. The description on the conduct of the study is very comprehensive and highly appreciated. However, based on the title of this manuscript and the introduction, I was expecting that the findings from the 4 sites would be presented and any dissimilar findings regarding Dementia would be explained after it had been verified that Polit and Beck’s criteria of generalisability had been followed.

 I would like to suggest the change in the title “Assessing the generalisability of a multicentre qualitative dementia research: the experience and challenges faced by the MinD project in Europe”

Is the rationale for developing the new method (or application) clearly explained?

Yes

Is the description of the method technically sound?

Yes

Are the conclusions about the method and its performance adequately supported by the findings presented in the article?

Yes

If any results are presented, are all the source data underlying the results available to ensure full reproducibility?

Yes

Are sufficient details provided to allow replication of the method development and its use by others?

Yes

Reviewer Expertise:

Public Health – Health Policy and Health Economics

I confirm that I have read this submission and believe that I have an appropriate level of expertise to confirm that it is of an acceptable scientific standard, however I have significant reservations, as outlined above.

# Response

**Published:** 2021-09-06

**Authors:** Jennifer NW Lim

**Affiliations:** 119616, UK

Dear reviewer,

 We thank you for your kind feedback and have revised the title of the paper as suggested, to reflect its contents. 

 Thank you. On behalf of the authors, Dr. Lim
